# An exposure–safety analysis to support the dosage of the novel AKT inhibitor capivasertib

**DOI:** 10.1007/s00280-025-04775-8

**Published:** 2025-03-28

**Authors:** Carlos Fernandez Teruel, Marie Cullberg, Ignacio González-García, Gaia Schiavon, Diansong Zhou

**Affiliations:** 1 Clinical Pharmacology & Quantitative Pharmacology, Clinical Pharmacology & Safety Sciences, BioPharmaceuticals R&D, AstraZeneca, Cambridge, UK; 2 Clinical Pharmacology & Quantitative Pharmacology, Clinical Pharmacology & Safety Sciences, BioPharmaceuticals R&D, AstraZeneca, Gothenburg, Sweden; 3 Late Development Oncology, Oncology R&D, AstraZeneca, Cambridge, UK; 4 Clinical Pharmacology & Quantitative Pharmacology, Clinical Pharmacology & Safety Sciences, BioPharmaceuticals R&D, AstraZeneca, Waltham, MA USA

**Keywords:** AKT inhibitor, Capivasertib, Exposure–safety analysis, Exposure–response analysis, Modeling, Recommended dose

## Abstract

**Purpose:**

This study aimed to evaluate capivasertib exposure–response relationships for clinical safety events to support dosage selection.

**Methods:**

Data from 277 patients with solid tumors participating in three phase 1 studies were analyzed. Capivasertib 80–800 mg was administered as monotherapy orally twice daily (BID) on continuous or intermittent (4 days on, 3 days off [4/3] or 2 days on, 5 days off [2/5]) schedules. Relationships between exposure related metrics (dose, weekly dose, AUC, AUC_PWD_, C_max_, and C_min_) and probability of safety endpoints (adverse event [AE] leading to dose discontinuation, AE leading to dose modification, serious AE [SAE], AE grade ≥ 3, AE grade ≥ 1, diarrhea AE grade ≥ 2, rash AE grade ≥ 2, hyperglycemia AE grade ≥ 3 and increased blood glucose > 13.9 mmol/L) were evaluated by logistic regression.

**Results:**

Significant exposure–response relationships were identified for all safety endpoints evaluated, except for AE grade ≥ 1. The analysis suggested that most of the safety endpoints are driven by the total weekly exposure, whereas glucose elevations are driven by the exposure achieved within a dosing interval. The probability of experiencing an AE leading to dose discontinuation, AE leading to dose modification, SAE, AE grade ≥ 3, diarrhea or rash were lower with the 480 mg BID [4/3] schedule than with the 320 mg BID continuous schedule.

**Conclusion:**

Significant exposure–response relationships were identified for safety endpoints when capivasertib was administered to patients with solid tumors suggesting that the intermittent [4/3] schedule is better tolerated than the continuous schedule due to lower total weekly exposure.

**Supplementary Information:**

The online version contains supplementary material available at 10.1007/s00280-025-04775-8.

## Introduction

The phosphatidylinositol 3-kinase (PI3K)/Akt serine/threonine kinase (AKT) signaling pathway is an important signaling pathway regulating cell proliferation and survival [1, 2]. Hyperactivation of the pathway due to PTEN deficiency or activating mutations in the catalytic subunit alpha of phosphatidylinositol-3-kinase (*PIK3CA*) and in *AKT1* is implicated in tumor growth across cancer indications [3]; hence, the PI3K/AKT pathway has been a target of oncology drug discovery [1, 2, 4].

Capivasertib, a potent, selective inhibitor of all AKT isoforms (AKT1/2/3) [5, 6], demonstrated efficacy in combination with fulvestrant for the treatment of hormone receptor (HR)-positive/human epidermal growth factor receptor 2 (HER2)-negative breast cancer and with paclitaxel for the treatment of triple-negative breast cancer (TNBC) in clinical phase 2 trials with an intermittent capivasertib dose regimen of 400 mg twice daily (BID) given 4 days on, 3 days off [4/3] [7, 8]. The [4/3] schedule of capivasertib administration has been used in all subsequent phase 3 trials. The phase 3 CAPItello-291 trial in patients with aromatase inhibitor-resistant, HR-positive/HER2-negative advanced breast cancer showed statistically significant and clinically meaningful improvement in progression-free survival in the overall population and in the population of patients with *PIK3CA/AKT1/PTEN*-altered tumors [9]. These data led to approval of capivasertib–fulvestrant in several markets [10–15] and the inclusion of the combination as a treatment option in clinical guidelines [16, 17].

Earlier on during clinical development, the first-in-human study of capivasertib explored a range of oral doses (80–800 mg BID) and schedules (continuous, intermittent [4/3], and intermittent 2 days on, 5 days off [2/5]) for capivasertib given as monotherapy to patients with solid tumors [18, 19]. The recommended phase 2 doses were determined at 320 mg BID, 480 mg BID, and 640 mg BID for the continuous, [4/3], and [2/5] schedules, respectively, and were primarily based on empirical evaluation of dose-limiting toxicities (DLTs). The DLTs were diarrhea, rash, and hyperglycemia [18, 19], which were broadly consistent with observations from studies of other AKT inhibitors (ipatasertib and MK2206) [20, 21] and other drugs affecting the PI3K/AKT pathway [22]. Based on the combination of tolerability, pharmacokinetic (PK), and pharmacodynamic considerations, capivasertib 480 mg BID [4/3] was selected as the recommended phase 2 dose for capivasertib monotherapy [18]. There was also evidence of greater target engagement in tumor tissue with 480 mg BID on a [4/3] schedule than with 320 mg continuous dosing [18], further supporting dose and schedule selection. Findings from phase 1/2 trials reinforced selection of a slightly lower dose of 400 mg BID [4/3] when capivasertib is used in combination with fulvestrant or paclitaxel [7, 8, 23–25].

The PK of capivasertib have been characterized in studies of patients with solid tumors [18, 19, 23, 26, 27], in healthy volunteer studies [28–30] and in a population PK (PopPK) analysis of pooled studies [31]. Capivasertib plasma exposure was approximately dose proportional in the dose range of 80–480 mg, with a half-life of approximately 8 h [31]. Capivasertib PK showed moderate between-subject variability, and no patient covariate (including race, gender, body weight, renal or hepatic function) was predicted to impact exposure to capivasertib by > 20% [31]. Therefore, no a priori dose adjustment is required for intrinsic patient factors [10].

Information collected from early phase clinical trials is used for initial assessment of drug safety profile over a wide dose range and estimation of a maximum tolerated dose. Uncertainty in safety evaluations, however, is a common problem in oncology drug development due to the small sample size and use of adaptive or complex study designs [32]. This may complicate the analysis of exposure–response for adverse events (AEs) and, therefore, the selection of the optimal therapeutic regimen for further clinical trials. In addition, interpretation of safety information generated in oncology trials is often confounded by differences in baseline patient characteristics, the between-subject variability of drug exposure, complex treatment schedules, toxicity-mediated dose reductions and extensive use of co-medications [32]. Multivariate exposure–safety analyses of individual patient data allow to delineate the impact of various factors on the observed safety profiles across clinical studies with different designs and various patient populations, guiding the selection of the therapeutic regimen for future trials. Such an approach was used for risk–benefit analyses of treatment with the AKT inhibitor ipatasertib in prostate cancer [33, 34], the programmed cell death ligand 1 antibody atezolizumab [35], the programmed cell death (PD-1) antibody nivolumab [36], and hypofractionated radiotherapy regimens [37], a model-based analysis was also applied to evaluate factors affecting the probability of immune-related AEs associated with the PD-1 antibody pembrolizumab [38].

The aim of the analysis reported here was to evaluate exposure–response relationships for the following safety endpoints of capivasertib when given as monotherapy to patients with solid tumors: AE leading to dose discontinuation, AE leading to dose modification (interruption and/or reduction), serious AE (SAE), AE grade ≥ 3, AE grade ≥ 1, diarrhea AE grade ≥ 2, rash AE grade ≥ 2, hyperglycemia AE grade ≥ 3 and increased blood glucose > 13.9 mmol/L, based on pooled data from three phase 1 clinical trials.

## Methods

### Experimental data

Safety data from 277 patients participating in three trials of capivasertib given as monotherapy were included in the analysis (Supplementary Table 1).

Study 1 (NCT01226316) was a phase 1 open-label multipart study. Parts A and B were dose-escalation and dose-expansion parts, respectively, to assess tolerability of capivasertib in patients with advanced solid malignancies; parts C and D were expansion cohorts of patients with *PIK3CA*-mutated breast or gynecologic cancers or *AKT1*-mutated breast or gynecologic cancers or other solid tumors to assess safety and efficacy of the selected dosing regimen [18, 25, 39]. The duration of the treatment cycles was 21 days. For parts A and B, the treatment was started with a single capivasertib administration, followed by a 3- to 7-day washout (Cycle 0). Patients received capivasertib 80–800 mg BID on a continuous or intermittent schedule [4/3] and [2/5] [18]. For parts C and D, capivasertib monotherapy was given at 480 mg [4/3] [18, 25, 39]. Part D also included patients receiving capivasertib in combination with fulvestrant [25], but only patients receiving capivasertib as monotherapy have been included in the present analysis.

Study 4 (NCT01353781) was a phase 1 dose-escalation study in Japanese patients with advanced solid malignancies [19]. The treatment was started with single capivasertib administration, followed by a 3- to 7-day washout (Cycle 0); duration of subsequent cycles was 21 days. Patients received capivasertib 80–640 mg BID on a continuous or intermittent schedule [4/3] and [2/5].

The OAK study (NCT01895946) was a phase 1 open-label study aimed to compare tablet and capsule capivasertib formulations and to explore the impact of food intake on drug exposure [26]. Patients received 480 mg capivasertib BID on an intermittent schedule [4/3].

### Dose and systemic exposure calculations

Blood samples for characterizing capivasertib PK were collected and analyzed using a PopPK approach [31], which was used to project the individual exposure metrics. The exposure–response analyses were conducted using a subset of patients from the PopPK analysis where capivasertib was used as monotherapy. The capivasertib dose, planned weekly dose (PWD), area under the curve (AUC) at steady-state (SS), AUC based on planned weekly dose (AUC_PWD_) at SS, maximum concentration (C_max_) at SS and minimum concentration (C_min_) at SS were selected as the exposure metrics for the exposure–response analyses. As different schedules were assessed between and within studies, the exposure metrics were derived for the last dose of the last dosing day in the second week of treatment (i.e. on days 14, 11, and 9 for the continuous, [4/3] and [2/5] schedules, respectively), to ensure SS conditions, using the planned dose. C_max_ was the maximum concentration, C_min_ was the 12 h post-dose concentration and AUC was calculated from dose/apparent clearance (CL/F). The relative dose intensity (RDI) was calculated as the percentage of the average weekly dose relative to the PWD to examine the tolerability of capivasertib monotherapy at the given dosing schedules.

### Safety endpoints

AEs were collected throughout the trials, graded using Common Terminology Criteria for Adverse Events v4. Treatment-emergent AEs (TEAEs), defined as any AE that started after the first dose of study treatment or that started prior to dosing and worsened during on treatment period (by investigator report of a change in intensity) following exposure to treatment, were included in the analysis.

The following safety endpoints were evaluated: AE leading to dose discontinuation, AE leading to dose modification (interruption and/or reduction), SAE, AE grade ≥ 3, AE grade ≥ 1, diarrhea AE grade ≥ 2, rash AE grade ≥ 2, hyperglycemia AE grade ≥ 3 and increased blood glucose > 13.9 mmol/L. An SAE was defined as any AE that resulted in death, was life-threatening, required inpatient hospitalization or prolongation of existing hospitalization, or resulted in a significant, persistent or permanent disability, or a congenital anomaly or birth defect in any subsequent children. Increased blood glucose was defined as > 13.9 mmol/L based on the Common Terminology Criteria for Adverse Events version 4 (CTCAE v4) criteria for grade 3 hyperglycemia used in the studies included in this analysis. These analyses were confined to hyperglycemia AE grade ≥ 3 and were not designed to assess the use of comedications during the treatment, such as hypoglycemic agents. The absolute value or percentage change to blood glucose were not included in the analyses. The rash AE endpoint pooled the following Medical Dictionary for Regulatory Activities (MedDRA) preferred terms: rash maculo-papular, rash macular, rash, rash generalized, rash papular and rash pruritic, while the diarrhea AE and hyperglycemia AE included the MedDRA preferred terms diarrhea and hyperglycemia, respectively.

The grade selection for diarrhea and rash was based on clinical relevance in terms of limiting quality of life; for hyperglycemia the grade was higher considering this is a pharmacodynamic effect of AKT inhibition and often seen as asymptomatic change of laboratory value only.

### Exposure–response analysis

Logistic regression models were used to establish associations between the probability of the safety endpoints with the exposure metrics at a statistically significant level of 0.005.

### Software

Dataset preparation was performed using Statistical Analysis Software (SAS^®^) version 9.4 (SAS Institute, NC, USA) and R Project for Statistical Computing, Version 4.0.2 (Comprehensive R Archive Network, http://cran.r-project.org) according to the data specifications. The analysis was performed using NONMEM Version 7.3.0 (ICON, Ellicot City, MD, USA), entimICE version 4.4 (Entimo AG, Berlin, Germany) and R Project for Statistical Computing.

## Results

### Patient characteristics and planned Capivasertib dose

Patient baseline characteristics are described in Table 1. Overall, the dataset comprised 277 subjects; 67.5% were females and 76.9% identified as White. The median (range) for weight was 70 (32–129) kg and 76.2% of the patients were < 65 years of age. All patients had advanced solid tumors and were classified as either 0 or 1 for WHO performance status. Primary tumor sites included breast, lung, cervix, uterus, pleura, ovary, liver, colorectal, or colon [18, 19, 26].

**Table 1:** Baseline patient characteristics

Characteristic		Study 1 (*N* = 206)	Study 4 (*N* = 41)	OAK (*N* = 30)	Total (*N* = 277)
Age, years; n (%)	≥ 65 < 65	52 (25.2) 154 (74.8)	4 (9.8) 37 (90.2)	10 (33.3) 20 (66.7)	66 (23.8) 211 (76.2)
Sex; n (%)	Female Male	148 (71.8) 58 (28.2)	24 (58.5) 17 (41.5)	15 (50.0) 15 (50.0)	187 (67.5) 90 (32.5)
Race	White Asian Japanese Other Asian Black Other Missing	185 (89.8) 9 (4.4) 3 (1.5) 6 (2.9) 5 (2.4) 5 (2.4) 2 (1.0)	0 41 (100) 41 (100) 0 0 0 0	28 (93.3) 0 0 0 1 (3.3) 1 (3.3) 0	213 (76.9) 50 (18.1) 44 (15.9) 6 (2.2) 6 (2.2) 6 (2.2) 2 (0.7)
Body weight, kg; median (range)		72 (32–129)	57 (40–97)	78 (53–115)	70 (32–129)
Hepatic function	Normal	150 (72.8)	32 (78.0)	22 (73.3)	204 (73.6)
	Mild impairment	53 (25.7)	9 (22.0)	8 (26.7)	70 (25.3)
	Moderate impairment	3 (1.5)	0 (0.0)	0 (0.0)	3 (1.1)
Renal function	Normal	119 (57.8)	28 (68.3)	20 (66.7)	167 (60.3)
	Mild impairment	70 (34.0)	9 (22.0)	10 (33.3)	89 (32.1)
	Moderate impairment	17 (8.3)	4 (9.8)	0 (0.0)	21 (7.6)
Blood glucose, mmol/L; median (range)		5.3 (3.7–8.1)	5.2 (4.6–7.2)	5.4 (3.7–6.5)	5.3 (3.7–8.1)^a^
HbA1c, mmol/mol; median (range)		37.0 (25.0–49.0)	46.0 (35.0–56.0)	36.0 (24.0–49.0)	37.0 (24.0–56.0)^b^

HbA1c, glycated hemoglobin

^a^Missing: 9 patients (3.2%); ^b^Missing: 49 patients (17.7%)

Capivasertib was administered as monotherapy at 80–800 mg BID, where 480 mg BID was the most common dose level (61.0% of patients; Supplementary Table 2), as it was the selected recommended phase 2 dose (given [4/3]) in the expansion phases of the dose-escalation studies and for the formulation/food study. The recommended doses of 320 mg for the continuous and 640 mg BID for the [2/5] schedule were less frequently used (6.5% and 8.7% of patients, respectively; Supplementary Table 2).

### Summary of exposure metrics and safety endpoints

The summary of the exposure metrics and safety endpoints by schedule and dose level are given in Tables 2 and 3, respectively.

The RDI was predominantly decreased with increasing dose for the continuous schedule and was ≤ 80% for the 400 and 480 mg doses. On the other hand, the RDI was > 90% for the [4/3] and [2/5] schedules at all dose levels, suggesting better tolerability with the intermittent schedules in patients enrolled in these early-phase studies (Table 2). In total, across all doses and schedules, 18.4% of patients had AEs leading to dose discontinuation, 50.2% AEs leading to dose modification, 38.6% SAEs, 65.7% AEs grade ≥ 3, 98.9% AE grade ≥ 1, 36.8% diarrhea AE grade ≥ 2, 26.4% rash AE grade ≥ 2, 26.0% hyperglycemia grade ≥ 3, and 45.5% increased blood glucose > 13.9 mmol/L (Table 3).

**Table 2:** Summary of exposure metrics by study, schedule, and dose level

Study	Schedule	Dose (mg)	PWD (mg)	*N*	Exposure metrics; median (range)				
					RDI (%)	AUC_ss(0–12 h)_ (mg∙h/L)	AUC_PWD_ (mg∙h/L)	C_max_ (mg/L)	C_min_ (mg/L)
Study 1	Continuous	80	1120	5	100.0 (95.9–100.6)	0.87 (0.69–1.70)	12.16 (9.71–23.78)	0.15 (0.13–0.26)	0.03 (0.02–0.09)
		160	2240	5	97.6 (83.5–102.3)	2.99 (1.94–4.80)	41.79 (27.09–67.27)	0.42 (0.37–0.83)	0.11 (0.07–0.19)
		240	3360	6	100.3 (98.8–100.9)	3.19 (2.15–4.68)	44.70 (30.11–65.50)	0.53 (0.31–0.97)	0.11 (0.08–0.16)
		320	4480	12	98.1 (37.2–102.4)	5.86 (3.51–10.74)	82.01 (49.12–150.34)	0.87 (0.58–1.62)	0.20 (0.10–0.38)
		400	5600	11	80.3 (55.4–102.1)	5.79 (4.28–13.70)	81.04 (59.96–191.78)	0.90 (0.52–2.26)	0.19 (0.14–0.55)
		480	6720	6	50.2 (45.3–83.3)	9.98 (7.24–18.06)	139.62 (101.36–252.63)	1.62 (1.12–2.33)	0.37 (0.16–0.77)
		600	8400	2	103.1 (100–106.2)	14.27 (12.74–15.79)	199.70 (178.34–221.05)	2.52 (2.04–3.00)	0.46 (0.43–0.50)
	[4/3]	480	3840	127	97.9 (26.4–138.9)	9.69 (3.97–26.5)	77.42 (31.74–212.16)	1.59 (0.60–3.91)	0.33 (0.11–1.07)
		640	5120	10	100.0 (70.5–122.2)	15.92 (9.62–28.95)	127.41 (76.99–231.67)	2.43 (1.41–5.27)	0.55 (0.24–1.30)
	[2/5]	640	2560	8	94.0 (80.9–109.1)	14.96 (8.18–26.83)	59.82 (32.70–107.11)	2.08 (1.35–3.90)	0.57 (0.28–0.95)
		800	3200	14	101.1 (42.5–133.3)	26.44 (11.83–70.78)	105.81 (47.34–283.19)	3.93 (2.34–5.60)	1.08 (0.38–3.61)
Study 4	Continuous	80	1120	3	100.0 (88.7–102.3)	1.48 (1.17–2.40)	20.74 (16.33–33.63)	0.22 (0.14–0.49)	0.06 (0.05–0.08)
		240	3360	7	100.0 (41.9–106.7)	5.22 (2.71–10.71)	73.04 (37.97–150.00)	0.82 (0.56–1.67)	0.17 (0.08–0.46)
		320	4480	6	91.5 (59.3–101.1)	6.56 (4.15–28.42)	91.79 (58.03–396.46)	1.38 (0.74–3.99)	0.15 (0.12–1.40)
		400	5600	5	72.1 (51.4–85.7)	7.75 (6.91–13.91)	108.53 (96.72–194.44)	1.24 (1.06–2.35)	0.27 (0.21–0.55)
	[4/3]	360	2880	8	107.9 (91.4–112)	6.10 (3.95–11.18)	48.80 (31.58–89.44)	0.96 (0.53–1.84)	0.21 (0.10–0.47)
		480	3840	6	107.9 (79.1–108.6)	10.85 (7.97–12.05)	86.78 (63.79–96.48)	1.88 (1.55–2.78)	0.29 (0.19–0.42)
	[2/5]	640	2560	6	111.2 (100–119)	15.24 (13.39–34.02)	60.97 (53.56–136.17)	2.48 (1.61–3.40)	0.57 (0.40–1.65)
OAK	[4/3]	480	3840	30	100 (45.6–115.4)	9.75 (4.43–26.60)	77.97 (35.49–213.33)	1.58 (0.90–4.63)	0.33 (0.12–1.51)

AUC_ss(0–12 h)_, area under the curve during a dose interval (0–12 h) at steady-state; AUC_PWD_, area under the curve based on planned weekly dose; C_max_, maximum plasma concentration; C_min_, minimum plasma concentration; PWD, planned weekly dose; RDI, relative dose intensity

**Table 3:** Summary of safety endpoints by dosing schedule and dose level

Schedule	Dose (mg)	*N*	Safety endpoint; *n* (%)								
			AEDD	AEDM	AE grade ≥ 1	AE grade ≥ 3	Diarrhea AE grade ≥ 2	Increased BG > 13.9 mmol/L	Hyperglycemia AE grade ≥ 3	Rash AE grade ≥ 2	SAE
Continuous	80	8	0	2 (25.0)	8 (100)	2 (25.0)	0	0	0	1 (12.5)	2 (25.0)
	160	5	0	1 (20.0)	5 (100)	0	0	0	0	1 (20.0)	1 (20.0)
	240	13	1 (7.7)	2 (15.4)	12 (92.3)	4 (30.8)	4 (30.8)	4 (30.8)	2 (15.4)	3 (23.1)	0
	320	18	3 (16.7)	8 (44.4)	18 (100)	10 (55.6)	5 (27.8)	3 (16.7)	2 (11.1)	5 (27.8)	4 (22.2)
	400	16	7 (43.8)	15 (93.8)	16 (100)	15 (93.8)	10 (62.5)	7 (43.8)	4 (25.0)	8 (50.0)	6 (37.5)
	480	6	4 (66.7)	5 (83.3)	6 (100)	6 (100)	5 (83.3)	2 (33.3)	2 (33.3)	3 (50.0)	6 (100)
	600	2	2 (100)	1 (50.0)	2 (100)	2 (100)	1 (50.0)	1 (50.0)	0	2 (100)	2 (100)
[4/3]	360	8	0	3 (37.5)	6 (75.0)	3 (37.5)	4 (50.0)	3 (37.5)	1 (12.5)	1 (12.5)	0
	480	163	27 (16.6)	90 (55.2)	163 (100)	114 (69.9)	64 (39.3)	82 (50.3)	45 (27.6)	45 (27.6)	68 (41.7)
	640	10	4 (40)	2 (20.0)	10 (100)	7 (70.0)	3 (30.0)	7 (70.0)	4 (40.0)	0	6 (60.0)
[2/5]	640	14	2 (14.3)	2 (14.3)	14 (100)	11 (78.6)	3 (21.4)	8 (57.1)	7 (50.0)	2 (14.3)	7 (50.0)
	800	14	1 (7.1)	8 (57.1)	14 (100)	8 (57.1)	3 (21.4)	9 (64.3)	5 (35.7)	2 (14.3)	5 (35.7)
Total		277	51 (18.4)	139 (50.2)	274 (98.9)	182 (65.7)	102 (36.8)	126 (45.5)	72 (26.0)	73 (26.4)	107 (38.6)

AE, adverse event; AEDD, adverse event leading to dose discontinuation; AEDM, adverse event leading to dose modification (interruption and/or reduction); BG, blood glucose; SAE, serious AE

### Exposure–response relationships

Detailed exposure–response analysis was carried out to identify statistically significant relationships between exposure metrics and safety endpoints (Supplementary Fig. 1). No significant relationships were observed for AE grade ≥ 1. Significant relationships for the remaining safety endpoints are summarized below. The exposure metric that showed the most significant (lowest p-value) association with each safety endpoint (by univariate logistic regression) are highlighted below in bold:

- AE leading to dose discontinuation: **PWD**.
- AE leading to dose modification: PWD, AUC, **AUC**_**PWD**_, C_max_, and C_min_.
- AE grade ≥ 3: dose, PWD, AUC, **AUC**_**PWD**_, C_max_, and C_min_.
- SAE: dose, PWD, AUC, **AUC**_**PWD**_, C_max_, and C_min_.
- Hyperglycemia AE grade ≥ 3: dose, **AUC**, AUC_PWD_, C_max_, and C_min_.
- Increased blood glucose > 13.9 mmol/L: dose, AUC, AUC_PWD_, **C**_**max**_, and C_min_.
- Diarrhea AE grade ≥ 2: PWD, **AUC**_**PWD**_.
- Rash AE grade ≥ 2: **PWD**.

**Fig. 1:**
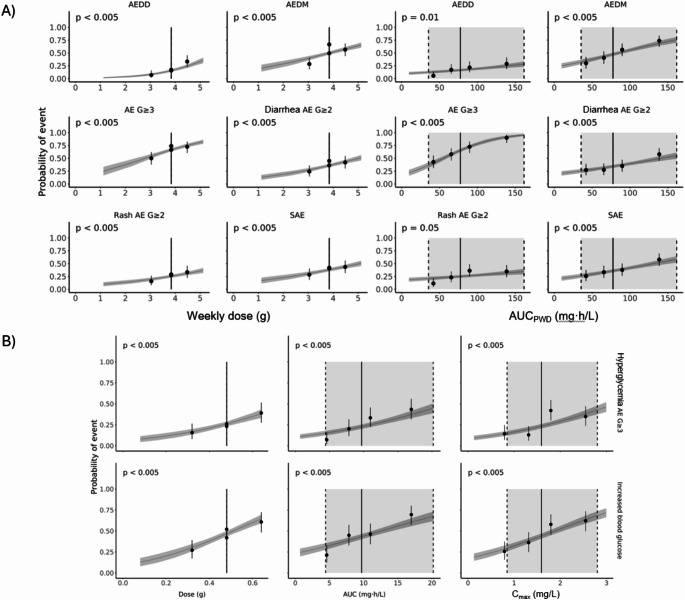
Relationship between exposure metrics and probability of event by (**A**) weekly dose and AUC_PWD_ and (**B**) Dose, AUC, and C_max_. Dots and solid black vertical lines: quartile of exposure metric with 95% CI; gray horizontal lines and dark gray areas: exposure–response relationship with 95% CI; solid and slashed gray vertical lines: 2.5th, 50th, and 97.5th percentiles of exposure metric at 480 mg [4/3]; light gray area: 95% prediction interval of exposure metric at 480 mg [4/3]; *p*-value represents the significance level of the exposure metric. AUC, area under the curve; AUC_PWD_, area under the curve based on planned weekly dose; AE, adverse event; AEDD, adverse event leading to dose discontinuation; AEDM, adverse event leading to dose modification (interruption and/or reduction); CI, confidence interval; C_max_, maximum plasma concentration; G, grade; SAE, serious adverse event

Hence, the total weekly exposure constituted the main driver for most safety endpoints (Fig. 1A), whereas hyperglycemia and increased blood glucose were primarily driven by the exposure achieved within the 12 h dosing interval (Fig. 1B).

**Fig. 2:**
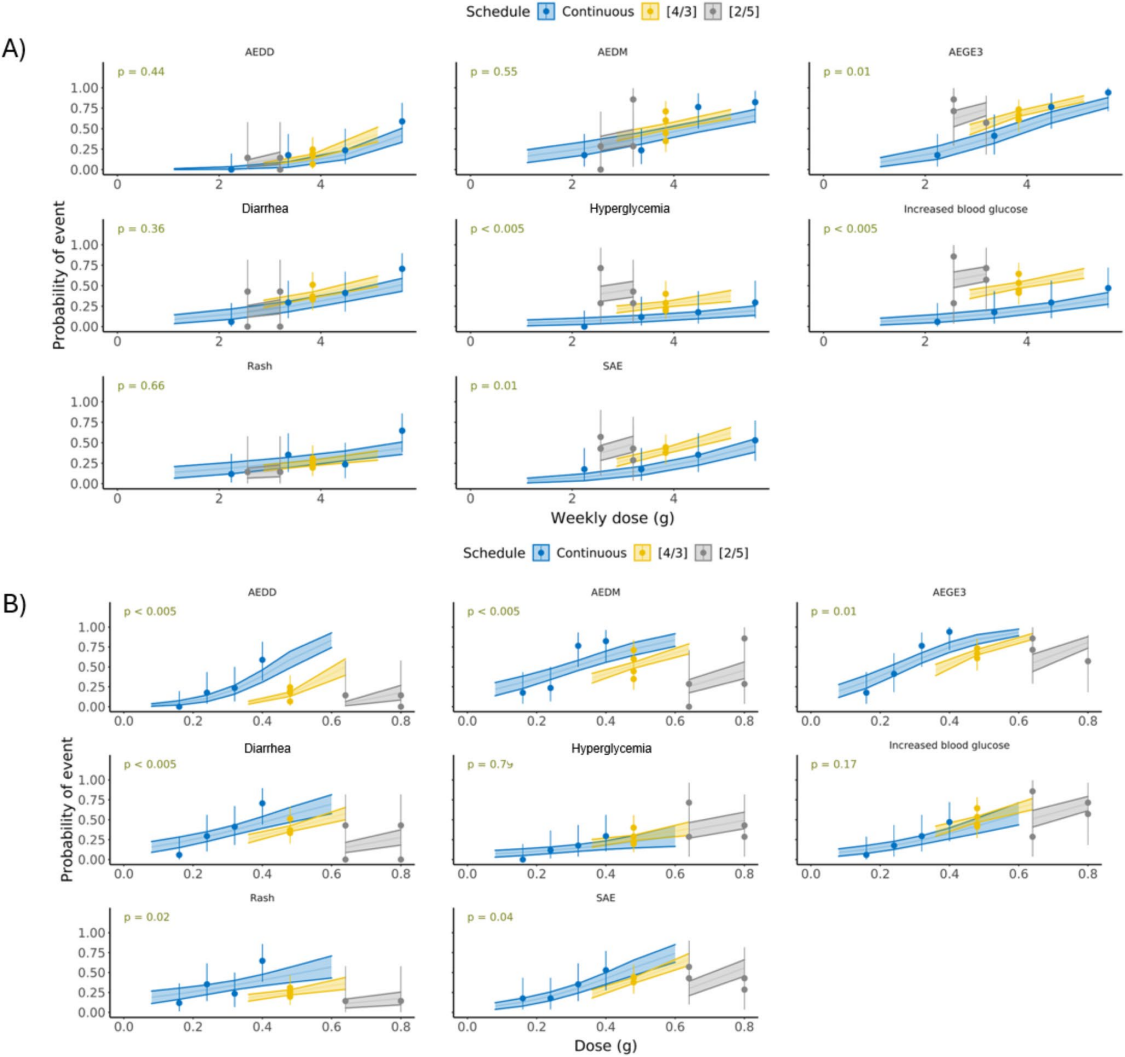
Relationship, according to administration schedule, between dose and probability of event by (**A**) dose and (**B**) weekly dose. Dots and vertical lines: quartile of exposure metric with 95% CI; horizontal lines and colored areas: exposure–response relationship with 95% CI; *p*-value represents the significance level of the schedule. AE, adverse event; AEDD, adverse event leading to dose discontinuation; AEDM, adverse event leading to dose modification (interruption and/or reduction); AEGE3, adverse event grade ≥ 3; CI, confidence interval; SAE, serious adverse event

At a given dose, there was a statistically significant effect of schedule for AE leading to dose discontinuation, AE leading to dose modification, and diarrhea AE grade ≥ 2 with intermittent schedules associated with lower probability of events compared with the continuous schedule, whereas the probabilities of hyperglycemia AE grade ≥ 3 and increased blood glucose > 13.9 mmol/L did not depend on the schedule (Fig. 2A). However, at a given weekly dose, the effect of schedule was statistically significant for hyperglycemia AE grade ≥ 3 and increased blood glucose > 13.9 mmol/L only, with intermittent schedules associated with higher probability of events compared with the continuous schedule; the [2/5] schedule was inferior, i.e. associated with higher probability of events, to the [4/3] schedule (Fig. 2B). Given the higher weekly dose on the 320 mg continuous schedule (4480 mg) compared with the intermittent schedule [4/3] at 480 mg (3840 mg), the probability of hyperglycemia AE grade ≥ 3 and increased blood glucose > 13.9 mmol/L is not expected to be significant between the continuous and intermittent 480 mg [4/3] schedules.

## Discussion

This safety analysis has characterized the exposure–response relationships for capivasertib monotherapy for AE leading to dose discontinuation, AE leading to dose modification, SAE, AE grade ≥ 3, diarrhea AE grade ≥ 2, rash AE grade ≥ 2, hyperglycemia AE grade ≥ 3, and increased blood glucose > 13.9 mmol/L with several exposure metrics. We focused on diarrhea, rash, and hyperglycemia in terms of AEs of special interest, as all three were DLTs as well as the most common AEs grade ≥ 3 in the first-in-human study of capivasertib (hyperglycemia was the most common, followed by diarrhea and then, maculopapular rash) [18]. The exposure–response models were able to establish relationships for all safety endpoints evaluated based on linear logistic regressions, except for AE grade ≥ 1, which occurred in almost all patients.

The exposure metrics were projected using the planned starting dose and proved to be adequate predictors for the incidence of the safety endpoints. Although other approaches, such as using the average exposure metric until the time of event, were considered, estimating different exposure metrics for each safety endpoint would complicate the subsequent interpretation as this may have led to biased analyses. Instead, the current analysis employed exposure metrics at SS, which are recommended as they do not depend on the outcome [40].

Hyperglycemia AE of grade ≥ 3 and increased blood glucose (> 13.9 mmol/L) were influenced by capivasertib exposure during a dosing interval, showing a stronger correlation with dose, AUC, and C_max_ than with the weekly exposure. Conversely, all other safety endpoints were predominantly associated with cumulative capivasertib exposure, demonstrating a stronger correlation with weekly dose and/or AUC_PWD_. Consequently, the likelihood of AE leading to dose discontinuation, AE leading to dose modification, SAE, AE of grade ≥ 3, diarrhea of grade ≥ 2, and rash of grade ≥ 2 is predicted to be lower with the [4/3] intermittent schedule compared with the continuous schedule at a given dose. However, the probability of hyperglycemia AE of grade ≥ 3 and increased blood glucose > 13.9 mmol/L is predicted to increase with the dose, regardless of the dosing schedule. While hyperglycemia is a known pharmacodynamic effect of AKT inhibition and often seen as asymptomatic change to blood glucose, oral hypoglycemic drugs are indicated in the clinical management of hyperglycemia AE grade ≥ 2. Use of metformin was explored at baseline, but usage was too low in the study population to allow meaningful inferences about combination therapy of capivasertib with hypoglycemic agents.

The probability of rash AE grade ≥ 2, AE leading to dose discontinuation, AE leading to dose modification, AE grade ≥ 3, SAE and diarrhea AE grade ≥ 2 was higher with the continuous schedule at 320 mg (4480 mg weekly) compared with the intermittent schedule [4/3] at 480 mg (3840 mg weekly) due to the lower total weekly exposure, while the [2/5] schedule at 640 mg presented higher probability of hyperglycemia AE grade ≥ 3 and increased blood glucose > 13.9 mmol/L than the [4/3] schedule at 480 mg. Therefore, our results further reinforce the selection of 480 mg BID on a [4/3] schedule as a recommended monotherapy dose based on safety outcomes.

## Conclusion

In patients with solid tumors who were administered capivasertib as monotherapy, significant relationships were identified between the total weekly exposure (weekly dose and AUC_PWD_) and the probability of experiencing AE leading to dose discontinuation, AE leading to dose modification (interruption and/or reduction), SAE, AE grade ≥ 3, diarrhea AE grade ≥ 2, and rash AE grade ≥ 2. The probabilities of experiencing hyperglycemia AE grade ≥ 3 and increased blood glucose > 13.9 mmol/L were more closely related to the exposure during a dosing interval (dose, AUC, and C_max_).

Overall, the analyses suggest that the 4/3 intermittent schedule was better tolerated, except for hyperglycemia AE grade ≥ 3 and increased blood glucose > 13.9 mmol/L events, compared with a continuous schedule due to the generally lower total weekly exposure.

## Electronic supplementary material

Below is the link to the electronic supplementary material.

Supplementary Material 1

Supplementary Material 2

## Data Availability

Data underlying the findings described in this manuscript may be obtained in accordance with the sponsor’s data sharing policy described at https://astrazenecagrouptrials.pharmacm.com/ST/Submission/Disclosure. Data can be requested through Vivli at https://vivli.org/members/enquiries-about-studies-not-listed-on-the-vivli-platform/. The sponsor’s Vivli member page is also available outlining further details: https://vivli.org/ourmember/astrazeneca/.

## References

[1] Zhang Y, Yan H, Xu Z, Yang B, Luo P, He Q (2019) Molecular basis for class side effects associated with PI3K/AKT/mTOR pathway inhibitors. Expert Opin Drug Metab Toxicol 15(9):767–774. 10.1080/17425255.2019.1663169 31478386 10.1080/17425255.2019.1663169

[2] He Y, Sun MM, Zhang GG, Yang J, Chen KS, Xu WW, Li B (2021) Targeting PI3K/Akt signal transduction for cancer therapy. Signal Transduct Target Ther 6(1):425. 10.1038/s41392-021-00828-5 34916492 10.1038/s41392-021-00828-5 PMC8677728

[3] Zhang Y, Kwok-Shing Ng P, Kucherlapati M, Chen F, Liu Y, Tsang YH, de Velasco G, Jeong KJ, Akbani R, Hadjipanayis A, Pantazi A, Bristow CA, Lee E, Mahadeshwar HS, Tang J, Zhang J, Yang L, Seth S, Lee S, Ren X, Song X, Sun H, Seidman J, Luquette LJ, Xi R, Chin L, Protopopov A, Westbrook TF, Shelley CS, Choueiri TK, Ittmann M, Van Waes C, Weinstein JN, Liang H, Henske EP, Godwin AK, Park PJ, Kucherlapati R, Scott KL, Mills GB, Kwiatkowski DJ, Creighton CJ (2017) A pan-cancer proteogenomic atlas of PI3K/AKT/mTOR pathway alterations. Cancer Cell 31(6):820–832e823. 10.1016/j.ccell.2017.04.013 28528867 10.1016/j.ccell.2017.04.013 PMC5502825

[4] Brown JS, Banerji U (2017) Maximising the potential of AKT inhibitors as anti-cancer treatments. Pharmacol Ther 172:101–115. 10.1016/j.pharmthera.2016.12.001 27919797 10.1016/j.pharmthera.2016.12.001 PMC6143165

[5] Addie M, Ballard P, Buttar D, Crafter C, Currie G, Davies BR, Debreczeni J, Dry H, Dudley P, Greenwood R, Johnson PD, Kettle JG, Lane C, Lamont G, Leach A, Luke RW, Morris J, Ogilvie D, Page K, Pass M, Pearson S, Ruston L (2013) Discovery of 4-amino-N-[(1S)-1-(4-chlorophenyl)-3-hydroxypropyl]-1-(7H-pyrrolo[2,3-d]pyrimidin– 4-yl)piperidine-4-carboxamide (AZD5363), an orally bioavailable, potent inhibitor of Akt kinases. J Med Chem 56(5):2059–2073. 10.1021/jm301762v 23394218 10.1021/jm301762v

[6] Davies BR, Greenwood H, Dudley P, Crafter C, Yu DH, Zhang J, Li J, Gao B, Ji Q, Maynard J, Ricketts SA, Cross D, Cosulich S, Chresta CC, Page K, Yates J, Lane C, Watson R, Luke R, Ogilvie D, Pass M (2012) Preclinical Pharmacology of AZD5363, an inhibitor of AKT: pharmacodynamics, antitumor activity, and correlation of monotherapy activity with genetic background. Mol Cancer Ther 11(4):873–887. 10.1158/1535-7163.Mct-11-0824-t 22294718 10.1158/1535-7163.MCT-11-0824-T

[7] Jones RH, Casbard A, Carucci M, Cox C, Butler R, Alchami F, Madden T-A, Bale C, Bezecny P, Joffe J, Moon S, Twelves C, Venkitaraman R, Waters S, Foxley A, Howell SJ (2020) Fulvestrant plus Capivasertib versus placebo after relapse or progression on an aromatase inhibitor in metastatic, oestrogen receptor-positive breast cancer (FAKTION): a multicentre, randomised, controlled, phase 2 trial. Lancet Oncol 21(3):345–357. 10.1016/S1470-2045(19)30817-4 32035020 10.1016/S1470-2045(19)30817-4 PMC7052734

[8] Schmid P, Abraham J, Chan S, Wheatley D, Brunt M, Nemsadze G, Baird R, Park YH, Hall P, Perren T, Stein RC, László M, Ferrero J-M, Phillips M, Conibear J, Sarker S-J, Prendergast A, Cartwright H, Mousa K, Turner NC (2018) AZD5363 plus Paclitaxel versus placebo plus Paclitaxel as first-line therapy for metastatic triple-negative breast cancer (PAKT): A randomised, double-blind, placebo-controlled, phase II trial. J Clin Oncol 36(15 Suppl):1007. 10.1200/JCO.2018.36.15_suppl.100729432078

[9] Turner NC, Oliveira M, Howell SJ, Dalenc F, Cortes J, Gomez Moreno HL, Hu X, Jhaveri K, Krivorotko P, Loibl S, Morales Murillo S, Okera M, Park YH, Sohn J, Toi M, Tokunaga E, Yousef S, Zhukova L, de Bruin EC, Grinsted L, Schiavon G, Foxley A, Rugo HS (2023) Capivasertib in hormone receptor-positive advanced breast cancer. N Engl J Med 388(22):2058–2070. 10.1056/NEJMoa2214131 37256976 10.1056/NEJMoa2214131 PMC11335038

[10] AstraZeneca Pharmaceuticals LP (2023) Highlights of prescribing information. TRUQAP™ (capivasertib) tablets, for oral use. https://www.accessdata.fda.gov/drugsatfda_docs/label/2023/218197s000lbl.pdf. Accessed December 18, 2024

[11] European Commission (2024) Union Register of medicinal products for human use. Truqap. https://ec.europa.eu/health/documents/community-register/html/h1820.htm. Accessed 2 September, 2024

[12] AstraZeneca Canada Inc (2024) Product monograph. Truqap, capivasertib tablets. https://pdf.hres.ca/dpd_pm/00074395.PDF. Accessed 2 September, 2024

[13] Australian Product Information, Truqap (2024) (capivasertib). https://www.ebs.tga.gov.au/ebs/picmi/picmirepository.nsf/pdf?OpenAgent=&id?CP-2024-PI-01864-1&d?20240612172310101&d?20240902172310101. Accessed 2 September, 2024

[14] AstraZeneca AG (2024) Truqap, Filmtabletten. 2024. https://www.swissmedicinfo.ch/ViewMonographie. Accessed 2 September, 2024

[15] Pharmaceuticals and Medical Devices Agency (2024) New Drugs Approved in FY 2023. https://www.pmda.go.jp/files/000269225.pdf. Accessed 2 September, 2024

[16] Cardoso F, Paluch-Shimon S, Schumacher-Wulf E, Matos L, Gelmon K, Aapro M, Bajpai J, Barrios C, Bergh J, Bergsten-Nordström E, Biganzoli L, Cardoso M, Carey L, Mac Gregor M, Chidebe R, Cortés J, Curigliano G, Dent R, El Saghir N, Eniu A, Fallowfield L, Francis P, Franco Millan S, Gilchrist J, Gligorov J, Gradishar W, Haidinger R, Harbeck N, Hu X, Kaur R, Kiely B, Kim S-B, Koppikar S, Kuper-Hommel M, Lecouvet F, Mason G, Mertz S, Mueller V, Myerson C, Neciosup S, Offersen B, Ohno S, Pagani O, Partridge A, Penault-Llorca F, Prat A, Rugo H, Senkus E, Sledge G, Swain S, Thomssen C, Vorobiof D, Vuylsteke P, Wiseman T, Xu B, Costa A, Norton L, Winer E (2024) 6th and 7th international consensus guidelines for the management of advanced breast cancer (ABC guidelines 6 and 7). Breast 76:103756. 10.1016/j.breast.2024.103756 38896983 10.1016/j.breast.2024.103756 PMC11231614

[17] Burstein HJ, DeMichele A, Fallowfield L, Somerfield MR, Henry NL, Testing ftB, Endocrine, Panels TTMBCE, Henry NL, Dayao Z, Elias A, Kalinsky K, McShane LM, Moy B, Park BH, Shanahan KM, Sharma P, Shatsky R, Stringer-Reasor E, Telli M, Turner NC, DeMichele A, Burstein HJ, Barton DL, Dorris A, Fallowfield LJ, Jain D, Johnston SRD, Korde LA, Litton JK, Macrae ER, Peterson LL, Vikas P, Yung RL, Rugo HS (2024) Endocrine and targeted therapy for hormone receptor–positive, human epidermal growth factor receptor 2–negative metastatic breast cancer—capivasertib-fulvestrant: ASCO rapid recommendation update. J Clin Oncol 42(12):1450–1453. 10.1200/jco.24.00248 38478799 10.1200/JCO.24.00248

[18] Banerji U, Dean EJ, Pérez-Fidalgo JA, Batist G, Bedard PL, You B, Westin SN, Kabos P, Garrett MD, Tall M, Ambrose H, Barrett JC, Carr TH, Cheung SYA, Corcoran C, Cullberg M, Davies BR, de Bruin EC, Elvin P, Foxley A, Lawrence P, Lindemann JPO, Maudsley R, Pass M, Rowlands V, Rugman P, Schiavon G, Yates J, Schellens JHM (2018) A phase I open-label study to identify a dosing regimen of the pan-AKT inhibitor AZD5363 for evaluation in solid tumors and in *PIK3CA*-mutated breast and gynecologic cancers. Clin Cancer Res 24(9):2050–2059. 10.1158/1078-0432.Ccr-17-2260 29066505 10.1158/1078-0432.CCR-17-2260

[19] Tamura K, Hashimoto J, Tanabe Y, Kodaira M, Yonemori K, Seto T, Hirai F, Arita S, Toyokawa G, Chen L, Yamamoto H, Kawata T, Lindemann J, Esaki T (2016) Safety and tolerability of AZD5363 in Japanese patients with advanced solid tumors. Cancer Chemother Pharmacol 77(4):787–795. 10.1007/s00280-016-2987-9 26931343 10.1007/s00280-016-2987-9 PMC4819940

[20] Xing Y, Lin NU, Maurer MA, Chen H, Mahvash A, Sahin A, Akcakanat A, Li Y, Abramson V, Litton J, Chavez-MacGregor M, Valero V, Piha-Paul SA, Hong D, Do K-A, Tarco E, Riall D, Eterovic AK, Wulf GM, Cantley LC, Mills GB, Doyle LA, Winer E, Hortobagyi GN, Gonzalez-Angulo AM, Meric-Bernstam F (2019) Phase II trial of AKT inhibitor MK-2206 in patients with advanced breast cancer who have tumors with *PIK3CA* or *AKT* mutations, and/or PTEN loss/*PTEN* mutation. Breast Cancer Res 21(1):78. 10.1186/s13058-019-1154-8 31277699 10.1186/s13058-019-1154-8 PMC6612080

[21] Sternberg CN, Bracarda S, de Bono JS, Sweeney C, Chi KN, Olmos D, Sandhu SK, Massard C, Garcia J, Schenkel F, Chen G, Harris A, Hinton H, Matsubara N (2021) 585P safety analysis of the phase III IPATential150 trial of Ipatasertib (ipat) plus abiraterone (abi) in patients with metastatic castration-resistant prostate cancer (mCRPC). Ann Oncol 32:S635–S636. 10.1016/j.annonc.2021.08.1098

[22] Rugo HS, André F, Yamashita T, Cerda H, Toledano I, Stemmer SM, Jurado JC, Juric D, Mayer I, Ciruelos EM, Iwata H, Conte P, Campone M, Wilke C, Mills D, Lteif A, Miller M, Gaudenzi F, Loibl S (2020) Time course and management of key adverse events during the randomized phase III SOLAR-1 study of PI3K inhibitor Alpelisib plus fulvestrant in patients with HR-positive advanced breast cancer. Ann Oncol 31(8):1001–1010. 10.1016/j.annonc.2020.05.001 32416251 10.1016/j.annonc.2020.05.001

[23] Turner NC, Alarcón E, Armstrong AC, Philco M, López Chuken YA, Sablin MP, Tamura K, Gomez Villanueva A, Perez-Fidalgo JA, Cheung SYA, Corcoran C, Cullberg M, Davies BR, de Bruin EC, Foxley A, Lindemann JPO, Maudsley R, Moschetta M, Outhwaite E, Pass M, Rugman P, Schiavon G, Oliveira M (2019) BEECH: a dose-finding run-in followed by a randomised phase II study assessing the efficacy of AKT inhibitor Capivasertib (AZD5363) combined with Paclitaxel in patients with Estrogen receptor-positive advanced or metastatic breast cancer, and in a PIK3CA mutant sub-population. Ann Oncol 30(5):774–780. 10.1093/annonc/mdz086 30860570 10.1093/annonc/mdz086 PMC6551452

[24] Howell SJ, Casbard A, Carucci M, Ingarfield K, Butler R, Morgan S, Meissner M, Bale C, Bezecny P, Moon S, Twelves C, Venkitaraman R, Waters S, de Bruin EC, Schiavon G, Foxley A, Jones RH (2022) Fulvestrant plus Capivasertib versus placebo after relapse or progression on an aromatase inhibitor in metastatic, oestrogen receptor-positive, HER2-negative breast cancer (FAKTION): overall survival, updated progression-free survival, and expanded biomarker analysis from a randomised, phase 2 trial. Lancet Oncol 23(7):851–864. 10.1016/S1470-2045(22)00284-4 35671774 10.1016/S1470-2045(22)00284-4 PMC9630162

[25] Smyth LM, Tamura K, Oliveira M, Ciruelos EM, Mayer IA, Sablin MP, Biganzoli L, Ambrose HJ, Ashton J, Barnicle A, Cashell DD, Corcoran C, de Bruin EC, Foxley A, Hauser J, Lindemann JPO, Maudsley R, McEwen R, Moschetta M, Pass M, Rowlands V, Schiavon G, Banerji U, Scaltriti M, Taylor BS, Chandarlapaty S, Baselga J, Hyman DM (2020) Capivasertib, an AKT kinase inhibitor, as monotherapy or in combination with fulvestrant in patients with AKT1 (E17K)-mutant, ER-positive metastatic breast cancer. Clin Cancer Res 26(15):3947–3957. 10.1158/1078-0432.Ccr-19-3953 32312891 10.1158/1078-0432.CCR-19-3953 PMC7415507

[26] Dean E, Banerji U, Schellens JHM, Krebs MG, Jimenez B, van Brummelen E, Bailey C, Casson E, Cripps D, Cullberg M, Evans S, Foxley A, Lindemann J, Rugman P, Taylor N, Turner G, Yates J, Lawrence P (2018) A phase 1, open-label, multicentre study to compare the capsule and tablet formulations of AZD5363 and explore the effect of food on the Pharmacokinetic exposure, safety and tolerability of AZD5363 in patients with advanced solid malignancies: OAK. Cancer Chemother Pharmacol 81(5):873–883. 10.1007/s00280-018-3558-z 29541803 10.1007/s00280-018-3558-z PMC5907623

[27] Miller C, Sommavilla R, O’Bryant CL, Barve M, Dowlati A, Luke JJ, Khatun M, Morris T, Cullberg M (2024) Pharmacokinetic study of Capivasertib and the CYP3A4 substrate Midazolam in patients with advanced solid tumors. Cancer Chemother Pharmacol 94(2):223–235. 10.1007/s00280-024-04667-3 38643311 10.1007/s00280-024-04667-3 PMC11390765

[28] Miller C, Sommavilla R, Barry ST, Eberlein C, Morris T, Wadsworth I, Cullberg M (2023) Pharmacokinetics of the Akt serine/threonine protein kinase inhibitor, Capivasertib, administered to healthy volunteers in the presence and absence of the CYP3A4 inhibitor Itraconazole. Clin Pharmacol Drug Dev 12(9):856–862. 10.1002/cpdd.1307 37449963 10.1002/cpdd.1307

[29] Miller C, Sommavilla R, Murphy D, Morris T, Khatun M, Cullberg M (2023) The effect of food and acid-reducing agents on the Pharmacokinetic profile of Capivasertib: results from a randomised, cross-over study. Br J Clin Pharmacol 89(11):3330–3339. 10.1111/bcp.15831 37328269 10.1111/bcp.15831

[30] Miller C, Wild M, Zhang Z, Sommavilla R, Shanahan D, Bailey C, Gränfors M, Bragg RA, Dong J, Sidhu S, Cullberg M (2024) A phase I study to determine the absolute bioavailability and absorption, distribution, metabolism, and excretion of Capivasertib in healthy male participants. Drug Metab Dispos 52(9):939–948. 10.1124/dmd.124.001636 39029948 10.1124/dmd.124.001636

[31] Fernandez-Teruel C, Cullberg M, Eberlein C, Barry ST, Zhou D (2024) Population pharmacokinetics of Capivasertib in patients with advanced or metastatic solid tumours. Clin Pharmacokinet 63(8):1191–1204. 10.1007/s40262-024-01407-x 39127854 10.1007/s40262-024-01407-x PMC11343776

[32] Ji Y, Jin JY, Hyman DM, Kim G, Suri A (2018) Challenges and opportunities in dose finding in oncology and immuno-oncology. Clin Transl Sci 11(4):345–351. 10.1111/cts.12540 29392871 10.1111/cts.12540 PMC6039198

[33] Zhu R, Poland B, Wada R, Liu Q, Musib L, Maslyar D, Cho E, Yu W, Ma H, Jin JY, Budha N (2019) Exposure–response-based product profile–driven clinical utility index for Ipatasertib dose selection in prostate cancer. CPT Pharmacometrics Syst Pharmacol 8(4):240–248. 10.1002/psp4.12394 30762302 10.1002/psp4.12394 PMC6482275

[34] Kotani N, Wilkins JJ, Wade JR, Dang S, Sutaria DS, Yoshida K, Sundrani S, Ding H, Garcia J, Hinton H, Sane R, Chanu P (2022) Characterization of exposure-response relationships of Ipatasertib in patients with metastatic castration-resistant prostate cancer in the IPATential150 study. Cancer Chemother Pharmacol 90(6):511–521. 10.1007/s00280-022-04488-2 36305957 10.1007/s00280-022-04488-2 PMC9637074

[35] Stroh M, Winter H, Marchand M, Claret L, Eppler S, Ruppel J, Abidoye O, Teng SL, Lin WT, Dayog S, Bruno R, Jin J, Girish S (2017) Clinical pharmacokinetics and pharmacodynamics of Atezolizumab in metastatic urothelial carcinoma. Clin Pharmacol Ther 102(2):305–312. 10.1002/cpt.587 27981577 10.1002/cpt.587

[36] Zhao X, Shen J, Ivaturi V, Gopalakrishnan M, Feng Y, Schmidt BJ, Statkevich P, Goodman V, Gobburu J, Bello A, Roy A, Agrawal S (2020) Model-based evaluation of the efficacy and safety of nivolumab once every 4 weeks across multiple tumor types. Ann Oncol 31(2):302–309. 10.1016/j.annonc.2019.10.015 31959348 10.1016/j.annonc.2019.10.015

[37] Thor M, Deasy JO, Paulus R, Robert Lee W, Amin MB, Bruner DW, Low DA, Shah AB, Malone SC, Michalski JM, Dayes IS, Seaward SA, Gore EM, Albert M, Pisansky TM, Faria SL, Chen Y, Koontz BF, Swanson GP, Pugh SL, Sandler HM (2019) Tolerance doses for late adverse events after hypofractionated radiotherapy for prostate cancer on trial NRG oncology/rtog 0415. Radiother Oncol 135:19–24. 10.1016/j.radonc.2019.02.014 31015166 10.1016/j.radonc.2019.02.014 PMC6582638

[38] Eun Y, Kim IY, Sun J-M, Lee J, Cha H-S, Koh E-M, Kim H, Lee J (2019) Risk factors for immune-related adverse events associated with anti-PD-1 pembrolizumab. Sci Rep 9(1):14039. 10.1038/s41598-019-50574-6 31575933 10.1038/s41598-019-50574-6 PMC6773778

[39] Hyman DM, Smyth LM, Donoghue MTA, Westin SN, Bedard PL, Dean EJ, Bando H, El-Khoueiry AB, Pérez-Fidalgo JA, Mita A, Schellens JHM, Chang MT, Reichel JB, Bouvier N, Selcuklu SD, Soumerai TE, Torrisi J, Erinjeri JP, Ambrose H, Barrett JC, Dougherty B, Foxley A, Lindemann JPO, McEwen R, Pass M, Schiavon G, Berger MF, Chandarlapaty S, Solit DB, Banerji U, Baselga J, Taylor BS (2017) AKT Inhibition in solid tumors with AKT1 mutations. J Clin Oncol 35(20):2251–2259. 10.1200/jco.2017.73.0143 28489509 10.1200/JCO.2017.73.0143 PMC5501365

[40] Wiens MR, French JL, Rogers JA (2024) Confounded exposure metrics. CPT Pharmacometrics Syst Pharmacol 13(2):187–191. 10.1002/psp4.13074 37984457 10.1002/psp4.13074 PMC10864924

