# Supplementary material for: An exposure–safety analysis to support the dosage of the novel AKT inhibitor capivasertib
Source: Cancer Chemother Pharmacol. 2025 Mar 28;95(1):48. doi: 10.1007/s00280-025-04775-8 (PMC11953117; doi:10.1007/s00280-025-04775-8)
Supplement: Supplementary file 1 — Supplementary Material 1 [file 280_2025_4775_MOESM1_ESM.docx]

# An exposure–safety analysis to support the dosage of the novel AKT inhibitor capivasertib

Carlos Fernandez Teruel^1^ • Marie Cullberg^2^ • Nacho Gonzalez^1^ • Gaia Schiavon^3^ • Diansong Zhou^4^

**Running head:** Capivasertib exposure–safety analysis

🖂 Diansong Zhou ([diansong.zhou@astrazeneca.com](mailto:diansong.zhou@astrazeneca.com))

**Author affiliations**

^1^Clinical Pharmacology & Quantitative Pharmacology, Clinical Pharmacology & Safety Sciences, BioPharmaceuticals R&D, AstraZeneca, Cambridge, United Kingdom

^2^Clinical Pharmacology & Quantitative Pharmacology, Clinical Pharmacology & Safety Sciences, BioPharmaceuticals R&D, AstraZeneca Gothenburg, Sweden

^3^Late Development Oncology, Oncology R&D, AstraZeneca, Cambridge, United Kingdom

^4^Clinical Pharmacology & Quantitative Pharmacology, Clinical Pharmacology & Safety Sciences, BioPharmaceuticals R&D, AstraZeneca Waltham, MA, United States of America

## Supplementary materials

### Supplementary Table 1 Experimental studies with safety information used in exposure calculations

| **Study** | **Phase** | **Design** | **Population** | **Capivasertib** | |
| --- | --- | --- | --- | --- | --- |
|  |  |  |  | **Dose** | **Schedule** |
| Study 1 [25,42,43] | 1 | Open-label, multicenter, adaptable dosing schedules | Patients with advanced solid tumors (*N* = 206) | Parts A and B: 80–800 mg BD | Parts A and B: Continuous or intermittent ([4/3] or [2/5]) |
|  |  |  |  | Parts C and D:  480 mg BD | Parts C and D:  Intermittent ([4/3]) |
| Study 4 [26] | 1 | Open-label, multicenter, adaptable dosing schedules | Japanese patients with advanced solid tumors (*N* = 41) | 80–640 mg BD | Continuous or intermittent ([4/3] or [2/5]) |
| OAK [31] | 1 | Open-label, multicenter, fixed-sequence, crossover | Patients with advanced solid tumors (*N* = 30) | 480 mg BD | Intermittent ([4/3]) |

BD, twice daily.

### Supplementary Table 2 Planned doses of capivasertib per study

| **Capivasertib planned dose BD (mg); n (%)** | **Study 1** **(*n* =** **206)** | **Study 4** **(*n* = 41)** | **OAK** **(*n* = 30)** | **Total** **(*N* =** **277)** |
| --- | --- | --- | --- | --- |
| 80 | 5 (2.4) | 3 (7.3) | 0 | 8 (2.9) |
| 160 | 5 (2.4) | 0 | 0 | 5 (1.8) |
| 240 | 6 (2.9) | 7 (17.1) | 0 | 13 (4.7) |
| 320 | 12 (5.8) | 6 (14.6) | 0 | 18 (6.5) |
| 360 | 0 | 8 (19.5) | 0 | 8 (2.9) |
| 400 | 11 (5.3) | 5 (12.2) | 0 | 16 (5.8) |
| 480 | 133 (64.6) | 6 (14.6) | 30 (100) | 169 (61.0) |
| 600 | 2 (1.0) | 0 | 0 | 2 (0.7) |
| 640 | 18 (8.7) | 6 (14.6) | 0 | 24 (8.7) |
| 800 | 14 (6.8) | 0 | 0 | 14 (5.1) |

BD, twice daily.

### Supplementary Fig. 1. Relationship between exposure metrics and safety endpoints


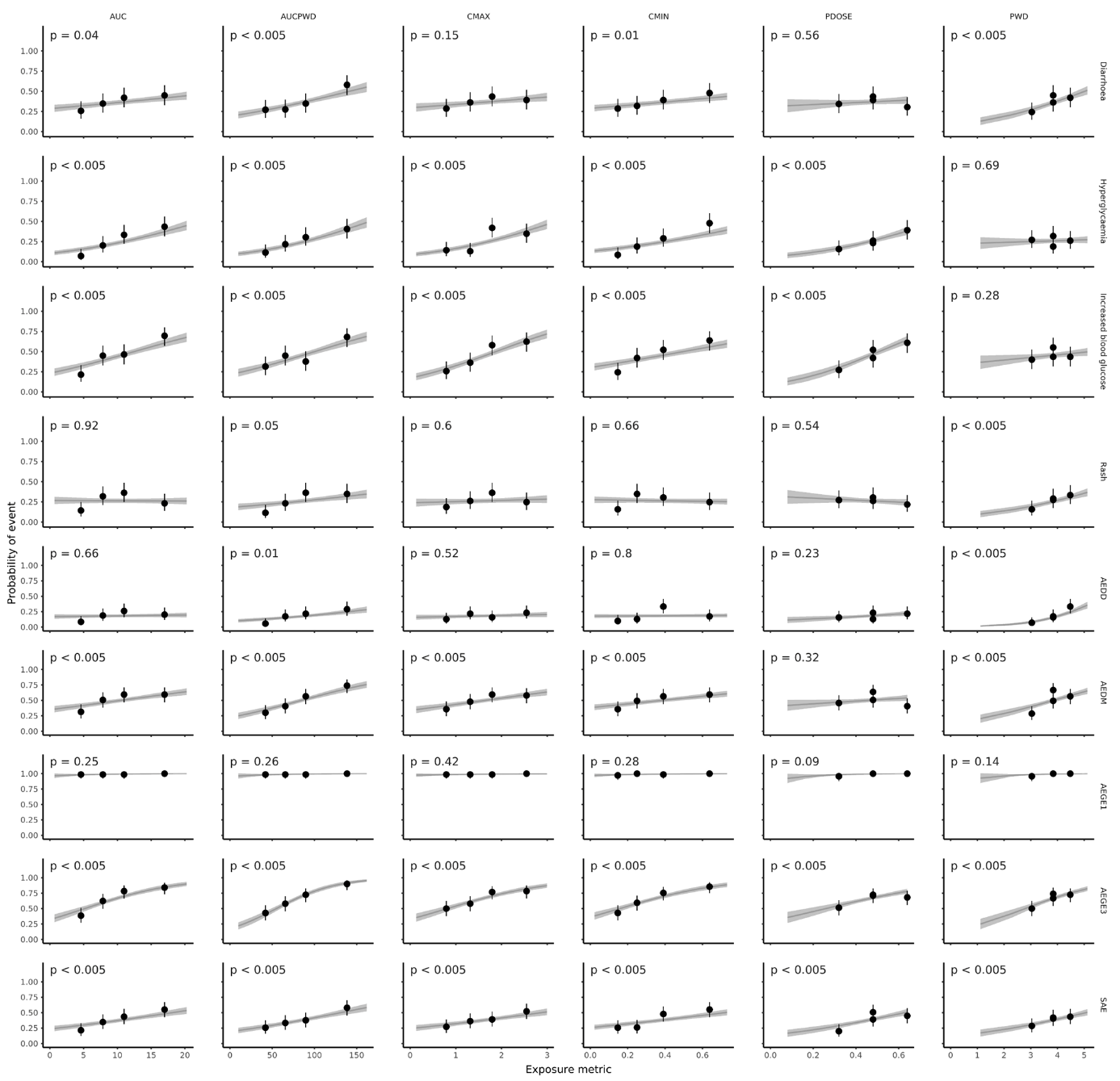


Dots and vertical lines: quartile of exposure metric with 95% CI; gray horizontal lines and gray area: exposure–response relationship with 95% CI; *p*-value represents the significance level of the exposure metric.

AUC, area under the curve; AUC_PWD_, area under the curve based on planned weekly dose; AE, adverse event; AEDD, adverse event leading to dose discontinuation; AEDM, adverse event leading to dose modification (interruption and/or reduction); AEGE1, AE grade ≥1; AEGE3, AE grade ≥3; CI, confidence interval; C_max_, maximum plasma concentration; C_min_, minimum plasma concentration; PWD, planned weekly dose; SAE, serious adverse event.
