# Supplementary figures and images for: An exposure–safety analysis to support the dosage of the novel AKT inhibitor capivasertib
Source: Cancer Chemother Pharmacol. 2025 Mar 28;95(1):48. doi: 10.1007/s00280-025-04775-8 (PMC11953117; doi:10.1007/s00280-025-04775-8)

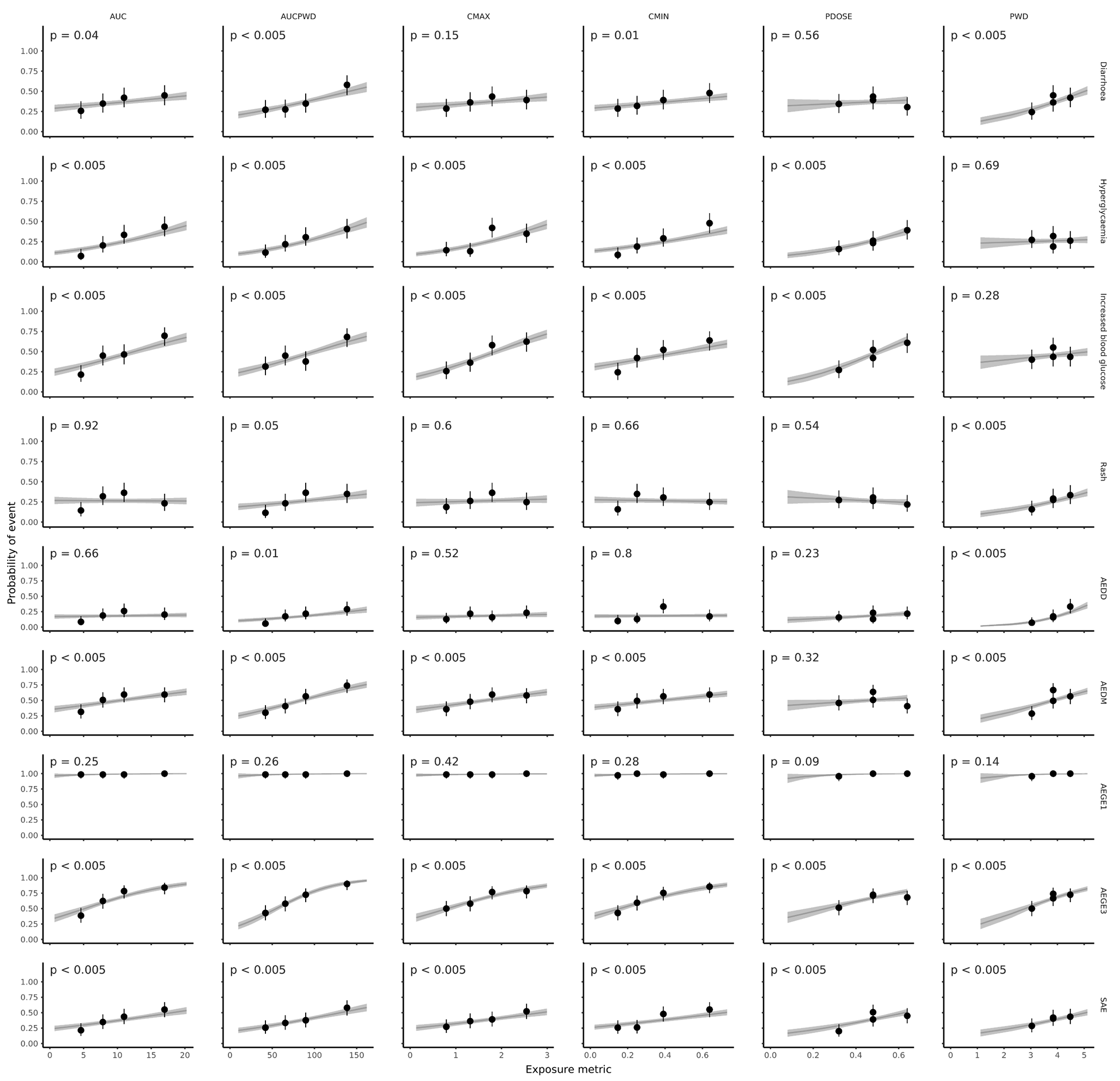

Supplement: Supplementary file 2 — Supplementary Material 2 [file 280_2025_4775_MOESM2_ESM.tif]
